# Rubbing Salt in the Wound: Molecular Evolutionary Analysis of Pain-Related Genes Reveals the Pain Adaptation of Cetaceans in Seawater

**DOI:** 10.3390/ani12243571

**Published:** 2022-12-16

**Authors:** Xiaoyue Ding, Fangfang Yu, Xiaofang He, Shixia Xu, Guang Yang, Wenhua Ren

**Affiliations:** Jiangsu Key Laboratory for Biodiversity and Biotechnology, College of Life Sciences, Nanjing Normal University, Nanjing 210000, China

**Keywords:** cetacean, pain, analgesia, relaxed selection, positive selection, molecular convergence

## Abstract

**Simple Summary:**

Cetaceans are aquatic mammals that evolved a series of specializations for life in an aquatic habitat, such as lack of distal hindlimbs, loss of hair, and derivation of echolocation. Notably, in the face of high salinity of seawater, the molecular mechanism of adaptation to pain in cetacean is still unclear. In this study, we performed molecular evolutionary analyses of genes related to pain perception (pain) and pain relief (analgesia) in selected representatives of mammals to explore the molecular mechanisms of pain adaptation in cetaceans to ‘rubbing salt in the wound’. Relaxed selection, positive selection, and convergent and specific amino acid substitutions were identified within cetacean’s pain-related genes, showing that the adaptation of mammals to a seawater environment might also include molecular evolution towards greater sensitivity to pain and more effective analgesia. Our study could have implications for diagnosis and treatment of human pain.

**Abstract:**

Pain, usually caused by a strong or disruptive stimulus, is an unpleasant sensation that serves as a warning to organisms. To adapt to extreme environments, some terrestrial animals have evolved to be inherently insensitive to pain. Cetaceans are known as supposedly indifferent to pain from soft tissue injury representatives of marine mammals. However, the molecular mechanisms that explain how cetaceans are adapted to pain in response to seawater environment remain unclear. Here, we performed a molecular evolutionary analysis of pain-related genes in selected representatives of cetaceans. *ASIC4* gene was identified to be pseudogenized in all odontocetes (toothed whales) except from *Physeter macrocephalus* (sperm whales), and relaxed selection of this gene was detected in toothed whales with pseudogenized *ASIC4*. In addition, positive selection was detected in pain perception (i.e., *ASIC3*, *ANO1*, *CCK*, and *SCN9A*) and analgesia (i.e., *ASIC3*, *ANO1*, *CCK*, and *SCN9A*) genes among the examined cetaceans. In this study, potential convergent amino acid substitutions within predicted proteins were found among the examined cetaceans and other terrestrial mammals, inhabiting extreme environments (e.g., V441I of TRPV1 in cetaceans and naked mole rats). Moreover, specific amino acid substitutions within predicted sequences of several proteins were found in the studied representatives of cetaceans (e.g., F56L and D163A of ASIC3, E88G of GRK2, and F159L of OPRD1). Most of the substitutions were located within important functional domains of proteins, affecting their protein functions. The above evidence suggests that cetaceans might have undergone adaptive molecular evolution in pain-related genes through different evolutionary patterns to adapt to pain, resulting in greater sensitivity to pain and more effective analgesia. This study could have implications for diagnosis and treatment of human pain.

## 1. Introduction

Pain is our body’s alarm system, warning us about dangers in the environment, wounds, and the presence of diseases. It is necessarily unpleasant, which enables protection by driving immediate attention, action, and adaptive learning [1]. Thus, the ability to detect harmful stimuli is critical for the survival of organisms. Pain is not only the result of a rigid hardware system, but also the interactions between highly plastic molecules and circuits [2]. Nociceptive stimuli that cause pain are sensed by special afferent nerve fibers called primary sensory neurons or nociceptors [3]. At the end of primary afferent neurons, four main nociceptors, i.e., acid-sensitive ion channels (ASICs), G protein-coupled receptors (GPCRs), P2X receptors (P2RXs), and transient receptor potential channels (TRP) are present [4,5]. In response to mechanical, thermal, and chemical stimuli, nociceptors cause membrane depolarization, which is called as generator potential. The initiated generator potentials are then converted into action potentials (AP) by ion channels, especially Nav1.7 channels, in adjacent areas. Next, the action potentials are transmitted further along the afferent sensory fibers wrapped by the epineurium. These fibers (whose body is located in the dorsal root ganglion) terminate in the spinal cord, where they communicate with the secondary neurons of the spinothalamic tract through synapses. Finally, the nociceptive signals are transmitted to the high-level brain center via the secondary neurons [6]. 

The pain response can be divided into physiological, psychological, and behavioral aspects, of which the latter one is the most easily to recognize, which is manifested as avoidance, crying, shouting, and so on [7,8,9]. However, during the process of adaptation to extreme environments some animals have lost their sense of pain or become insensitive to it in certain situations. For example, the African naked mole-rat (*Heterocephalus glaber*) acquired insensitivity to pain caused by acid stimuli. It was found that cutaneous nerves of the species are lacking in substance P (SP) and calcitonin gene-related peptide (CGRP) that are associated with nociceptive primary afferent fibers [10,11]. Another study indicated a mutation in TrkA gene as putative mechanism of pain insensitivity in the species [12]. Other example is pallid bat (*Antrozous pallidus*) that feeds on various species of scorpions. The species can endure multiple scorpion’s stings during hunting without behavioral pain response (e.g., avoidance and vocalizing) [13]. It was suggested that resistance to pain caused by scorpion venom might be acquired via amino acid substitutions in some proteins of Nav1.7 channel (e.g., E284G) [14]. 

So far, studies have investigated the mechanisms behind pain adaptation in mammals that are adapted to extreme terrestrial environments, but there are few studies on the mechanisms behind pain adaptation in marine mammals. Salinity is an important feature that distinguishes the marine from the terrestrial environment. The present in the saltwater sodium chloride, magnesium chloride and magnesium sulfate can directly stimulate the pain sensation in sea mammals when injury occurs [15]. In addition, salt has a strong water absorption function, which causes the edema around the wound to be more serious, further increasing the compression of pain nerves and causing acute pain [16]. Seals and sea lions that were heat-marked showed wound licking behavior and they stay in the water for a time about 12% shorter than before wounds acquisition [17,18]. It indicates that marine mammals can sense pain, and increased pain sensation of wounds caused by its contact with salt water. Moreover, bottlenose dolphins (*Tursiops 2runcates*) respond to painful stimuli with protest/withdrawal movements following a needle stick, suggesting that dolphins can perceive pain. However, bottlenose dolphins show apparent indifference to pain after soft tissue injury [19]. This also suggests that cetaceans may have adapted to pain in response to the marine environment. However, the molecular mechanism of this adaptation in cetaceans is still unclear.

At present, the most commonly used analgesia (the treatment of pain) methods are acupuncture and opioid therapy, which are mainly based on raising the pain threshold and attenuating/blocking pain signaling. In this study, we defined the genes reported to be involved in pain perception/sensation as pain genes or pain perception/sensation gene, and to be involved in analgesia as analgesia genes. The above pain perception/sensation and analgesia genes are collectively referred to as pain-related genes. We performed molecular evolutionary analyses of genes related to pain sensation and analgesia in representative mammals to explore the molecular mechanism of pain adaptation in cetaceans. This study could have implications for diagnosis and treatment of human pain.

## 2. Materials and Methods

### 2.1. Selected Species, Candidate Genes and Sequence Acquisition

Our research included the sequences of 31 pain-related genes, encompassing 18 pain perception genes: *SCN9A*, *SCN10A*, *PRDM12*, *ANO1*, *CCK*, *MARK1*, *MARK2*, *RUNX1*, *NGF*, *NTRK1*, *CACNA1G*, *TRPV1*, *TRPV4*, *P2RX3*, *P2RX4*, *ASIC1*, *ASIC3* and *ASIC4* and 13 analgesia genes: *CA8*, *GRK2*, *ARRB2*, *KCNA1*, *KCNA2*, *TRAAK*, *KCNIP3*, *KCNJ10*, *ANO3*, *OPRM1*, *OPRD1*, *OPRK1*, and *OPRL1*. All the selected genes have been proven to be associated with the pain (Supplementary Table S1 [20,21,22,23,24,25,26,27,28,29,30,31,32,33,34,35,36,37,38,39,40,41,42,43,44,45,46,47]). In total, 43 representatives of mammalian species were examined, including: 17 Cetacea, 4 Artiodactyla, 8 Carnivora, 2 Perissodactyla, 3 Chiroptera, 3 Primates, 1 Scandentia, 3 Rodentia, 1 Sirenia, and 1 Proboscidea. Most of the gene sequences were obtained from NCBI (http://www.ncbi.nlm.nih.gov/ accessed on 15 March 2022) and OrthoMaM [48] (https://orthomam.mbb.cnrs.fr/ accessed on 15 March 2022) (Supplementary Table S2). The gene sequences that are not available from the online databases, were extracted directly from well-annotated genomes stored in NCBI by our custom script. In addition, a set of genes of comparable size, 30 housekeeping genes (*ACTB*, *ACTG1*, *ARF4*, *ATP6V1F*, *BECN1*, *CALM2*, *EIF4A2*, *ERH*, *HBOA*, *HINT1*, *HPCAL1*, *ID3*, *LASP1*, *LDHA*, *NFKBIA*, *PABPC1*, *PGK1*, *RPL11*, *RPL32*, *RPLP1*, *RPS18*, *RPS19*, *RPS25*, *RPS27A*, *SEC61B*, *SEPTIN7*, *UBB*, *YWHAB*, *YWHAZ*, and *ZFP36L1*) of the above 43 species were used as a control set of genes to prove that the analysis results of pain-related genes are not randomly generated and are unusual. Housekeeping genes are being expressed stably in all conditions and cells, essentially, belong to cellular maintenance pathways and be conserved. Control genes sequences of human were downloaded from NCBI, and gene sequences of other species were blasted from genomes by using gene sequences of human as queries. The information of genomes was shown in Supplementary Table S3. The nucleotide and amino acid sequences (translated by MEGA) of each gene were aligned by MUSCLE in MEGA [49] and trimmed manually.

### 2.2. Screening for and Validating Pseudogenes

If premature stop codons, frame-shifting indels, and/or splicing site mutations were found in genes by alignment, then they were identified as potential pseudogenes and validated by PCR. Genomic DNA was extracted from cetacean muscle collected from baiji dolphin (*Lipotes vexillifer*) and Yangtze Finless Porpoise (*Neophocaena asiaeorientalis*) using the standard phenol/chloroform extraction method followed by ethanol precipitation [50]. All cetacean samples used in this study were collected from dead individuals in the wild with accordance to the ethical guidelines and legal requirements in China. DNA concentration and quality were measured by a UV spectrophotometer (Thermo Scientific of the U.S., NANODROP 2000). Primers (Supplementary Table S4) were designed in the conserved regions at both ends of the mutational locus in cetaceans by the online Primer-BLAST tool of NCBI. PCR amplification was performed according to the protocol of TIANGEN of the China (KT121221 2 × Taq Plus). PCR products were detected by agarose gel electrophoresis, and the product with the most distinct band was sent to Sangon Biotech for sequencing.

### 2.3. Selective Pressure Analysis

Comparison of ω (dN/dS), the ratio of nonsynonymous (dN) to synonymous (dS) substitution rates, among branches is an indication of the form and intensity of natural gene selection, where ω > 1, ω = 1 and ω < 1 indicate positive, neutral and purifying selection, respectively. The codon-based maximum likelihood models implemented in the CODEML program in PAML 4.7a [51] were employed to estimate the rates of synonymous (dS) and nonsynonymous substitutions (dN), as well as the dN/dS ratio (ω).

For pseudogenes, five models were used to determine the selection pressure in different branches. Model A assumes that all branches have the same ω values, model B is similar to model A, but ω is fixed to 1 (ω = 1). Model A versus model B was used to detect an overall selection pressure for pseudogenes. Model C assumes that particular pseudogenized branches have a common ω2, model D is similar to model C, but ω2 is fixed to 1 (ω2 = 1). Model C versus model D was used to detect the selection pressure for each pseudegenized branches. Finally, model E, which allowed different branches to have their own ω independently, was compared with model C to assess the divergence in the selective pressure across the phylogenetic branches [52].

In order to explore the evolutionary pattern of pain-related genes in cetaceans, we used the Branch Model and Branch-site Model in the PAML software package to perform selection pressure analysis on the selected genes. All the phylogenetic trees used in this study were obtained from the Timetree [53] website (http://www.timetree.org/ accessed on 20 March 2022). Foreground branches (tested as the pedigree being selected) and background branches (other pedigrees) are required to be defined a priori in the branch model and branch site model. The two-ratio model was treated as an alternative hypothesis for the branch model, which allows for the foreground and background branches have different ω values. The one-ratio model was used as the corresponding null hypothesis, which assumed that all evolutionary branches have the same ω value. In the branch-site model, the alternative hypothesis allowed each codon of the foreground branch to have its own ω value and allowed its ω to be greater than 1 (positive selection model: 0 < ω0 < 1, ω1 = 1 and ω2 ≥ 1); the null hypothesis (neutral model: 0 < ω0 < 1, ω1 = 1 and ω2 = 1) did not allow positive selection to appear. Then, we used the likelihood ratio test (LRT) with a χ^2^ distribution to determine which models were statistically different from the null model (threshold of *p* < 0.05). Moreover, the *p*-values of all genes were multiple-calibrated by false discovery rate (FDR) using the method of Benjamini–Hochberg [54], namely FDR (*p*^adjust^) < 0.05. Finally, we used Bayes empirical Bayes (BEB) analysis to determine sites under positive selection with posterior probabilities ≥ 0.8 [55].

### 2.4. Labeling Positive Selection Sites on the Three-Dimensional Structure of Proteins

To emphasize the significance of positively selected sites in function of protein, we mapped these sites onto the predicted 3D structure of proteins. First, we used protein sequences of the bottlenose dolphin to predict the 3D structure by I-TASSER (https://zhanglab.ccmb.med.umich.edu/services/ accessed on 5 April 2022). Then, we used the Pfam (http://pfam.xfam.org/ accessed on 8 April 2022) website to search for domains of each protein. Finally, the EzMol (http://www.sbg.bio.ic.ac.uk/ezmol/ accessed on 17 April 2022) was used to annotate the positively selective sites and functional domains in the obtained 3D structure. 

### 2.5. Identification of Convergent Amino Acids and Cetaceans-Specific Amino Acid Substitutions

To explore whether there are convergent molecular mechanisms among species that adapt to pain and whether cetaceans have their own unique molecular evolutionary mechanisms, we used MEGA 6 software and Alignment of Fasparser 2.10.0 software [56] to compare the amino acid sequences of pain-related genes, where set target group = 1, another group > 0.85, and ignore gaps. We defined that, for an amino acid site, if multiple species adapted to pain or cetaceans shared an amino acid that was unique to most other species, then that amino acid was convergent and related to pain insensitivity or cetaceans-specific amino acid substitutions.

### 2.6. Predicting the Effect of Cetacean-Specific Amino Acid Substitutions on Protein Function

We used Polyphen2 [57] (Phenotyping Version2, http://genetics.bwh.harvard.edu/pph2/ accessed on 5 May 2022) and PROVEAN [58] (Protein variation effect analyzer, http://provean.jcvi.org/seq_submit.php accessed on 5 May 2022) to predict the effect of cetacean-specific amino acid substitutions on protein function. The human protein sequences and cetacean-specific amino acid substitutions were inputted into the corresponding to protein sequences boxes and substitutions/mutation boxes of Polyphen2 and PROVEAN. The Polyphen2 score over the threshold is considered to affect protein function; a PROVEAN score less than or equal to the threshold −2.5 is considered deleterious (Deleterious), and a score higher than −2.5 is considered neutral, meaning the mutation may not affect the protein’s function.

## 3. Results

### 3.1. Pseudogenization of ASCI4 in Toothed Whales

**Figure 1:**
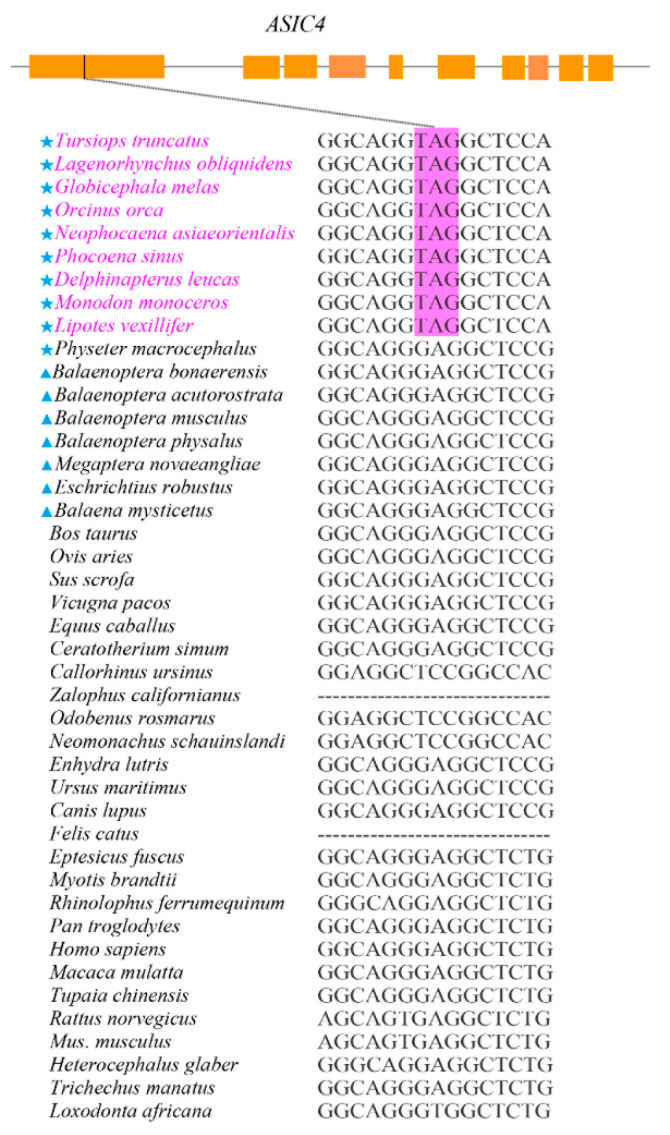
Pseudogenizations were detected in the *ASIC4* of almost all the toothed whales. A schematic diagram of the gene structure of *ASIC4* in the bottlenose dolphin is shown (**on the top**). Orange rectangles indicate exons and the black vertical bar indicates the location of premature stop codons. All 10 toothed whales in this study, except for sperm whales, had premature stop codons in the first exon of *ASIC4*. Blue stars represent toothed whale species; blue triangles represent baleen whale species. Purplish red font represents species with detected pseudogenes, and purplish red boxes are stop codons.

Of the 31 pain-related genes examined in this study, *ASCI4* was identified to be pseudogenized in all toothed whales except the sperm whale due to premature stop codons detected in the first exon of the sequences (Figure 1, Supplementary Figure S1). The other 30 genes were intact in all the cetaceans studied. In a control set of 30 housekeeping genes, we did not detect pseudogenization.

### 3.2. Relaxed Selection in ASIC4 Detected in Toothed Whales

Then, we calculated a series of ω values to determine whether the *ASIC4* gene has experienced relaxed selection pressure (Table 1). The average ω across the tree was estimated to be 0.087 in model A, which was significantly lower than in model B (ω = 1.000), indicating strong purifying selection on *ASIC4* (*p* = 0; Table 1). Model C allowed toothed whales with pseudogenized *ASIC4* having ω2 (ω_2_ = 0.200), fitting the data significantly better than model A (ω = 0.087; *p* = 0.004 in model C vs. A; Table 1). This indicates the relaxed selection pressure on ASIC4 gene in toothed whales. Model C (ω_2_ = 0.200) of the pseudogene branch was significantly different from model D (ω_2_ =1.000; *p* = 6.31221 × 10^−8^ in model C vs. D; Table 1), suggesting that the selective pressure on *ASIC4* was not completely relaxed. Finally, model E significantly fitted the data compared with model C (*p* = 6.15578 × 10^−12^ in model C vs. E; Table 1), indicating a divergence in the selective pressure across the tree.

### 3.3. Cetacean Lineages Displayed Stronger Positive Selection than Unreported Pain-Adaption Branches and Reported Pain-Adaption Non-Cetacean Branches

**Figure 2:**
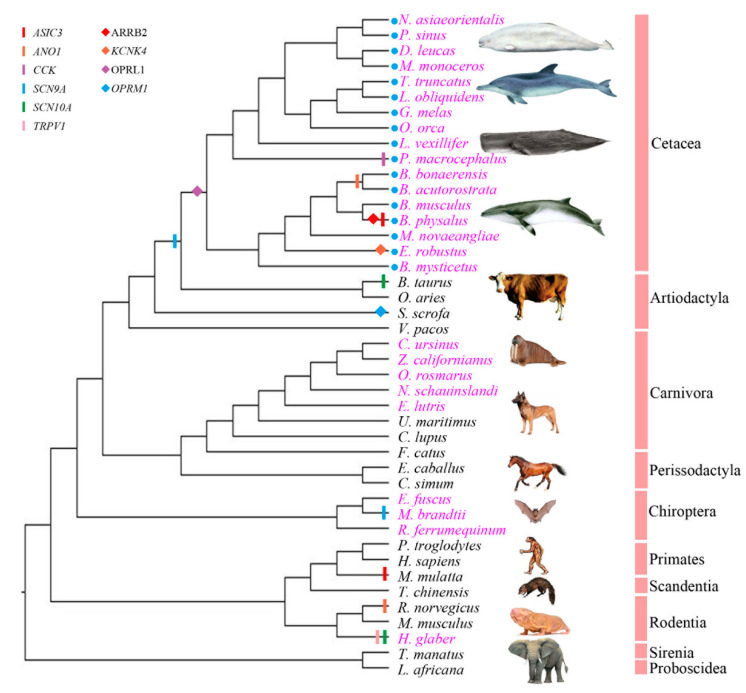
Positive selection detected in the mammalian dataset using the branch-site model. Blue circles represent cetacean species. Reported pain-adapted species are shown in purplish red font; unreported pain-adapted species are shown in black font. The lineages for which positive selection was detected on pain perception genes are shown with vertical bars of different colors: *ASIC3* (red), *ANO1* (orange), *CCK* (purple), *SCN9A* (blue), *SCN10A* (green), *TRPV1* (pink). The branches for which positive selection was detected on analgesia genes are indicated by diamonds of different colors: *ARRB2* (red), *KCNK4* (orange), *OPRL1* (purple), *OPRM1* (blue).

**Figure 3:**
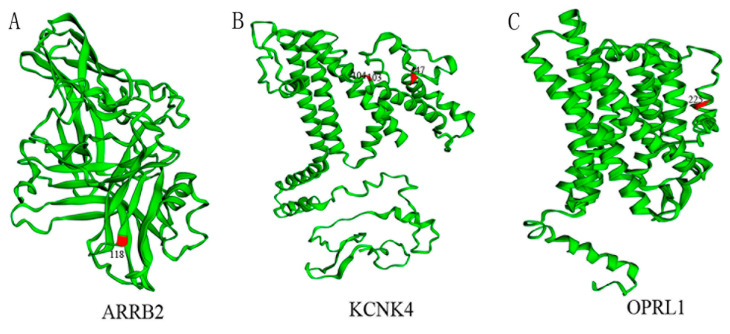
The distribution of positive selection sites on the three-dimensional structure. (**A**–**C**) represent the distribution of positive selection sites in ARRB2, KCNK4, and OPRL1, respectively. These genes were specifically detected positive selection signals in cetacean lineages. The yellow represents the domain located by positive selection sites, the purplish red represents the positive selection sites, and the corresponding numbers represent the amino acid positions.

Three positive selection instances in three genes (two pain perception genes, one analgesia gene) were detected in the mammalian dataset using the branch model and all were found in cetaceans, especially in baleen whales (Supplementary Table S5). In turns, ten positive selection instances in six pain perception genes and four positive selection instances in four analgesia genes were identified in the mammalian dataset using the branch-site model, when compared with a control set of housekeeping genes (no positive selection was detected). Among these 14 positive selection instances, cetacean lineages (7/14) displayed stronger positive selection than unreported pain-adaption branches (4/14) and reported pain-adaption non-cetacean branches (3/14) (Figure 2, Supplementary Table S6). In addition, positive selection with pain sensation genes were detected in selectively pain-insensitive species such as bats, naked mole rats, cows, and mice (for which no pain-related reports were reported), but they were mainly concentrated in cetacean branches (4/10), especially baleen whales and sperm whales (the only toothed whale with no pseudogene detected on *ASIC4*). Among the analgesia genes, we only detected positive selection signals in the cetacean lineages of three (*ARRB2*, *KCNK4*, and *OPRL1*), and positive selection signals were detected in *OPRM1* in pigs. In other words, analgesic genes (3/4) showed more signals in cetacean lineages than pain perception genes (4/10) (Figure 2, Supplementary Table S6). Furthermore, most positive selection sites were located at or near the protein’s functional domain. For example, the 118th position of the ARRB2 protein is located in the Arrestin_N domain; the 103rd and 104th positions of the KCNK4 protein are located in the Ion_trans_2 domain; the 225th position of the OPRL1 protein is located in the 7tm_1 domain (Figure 3). 

### 3.4. Potential Molecular Convergence among Species Adapted to Pain

**Figure 4:**
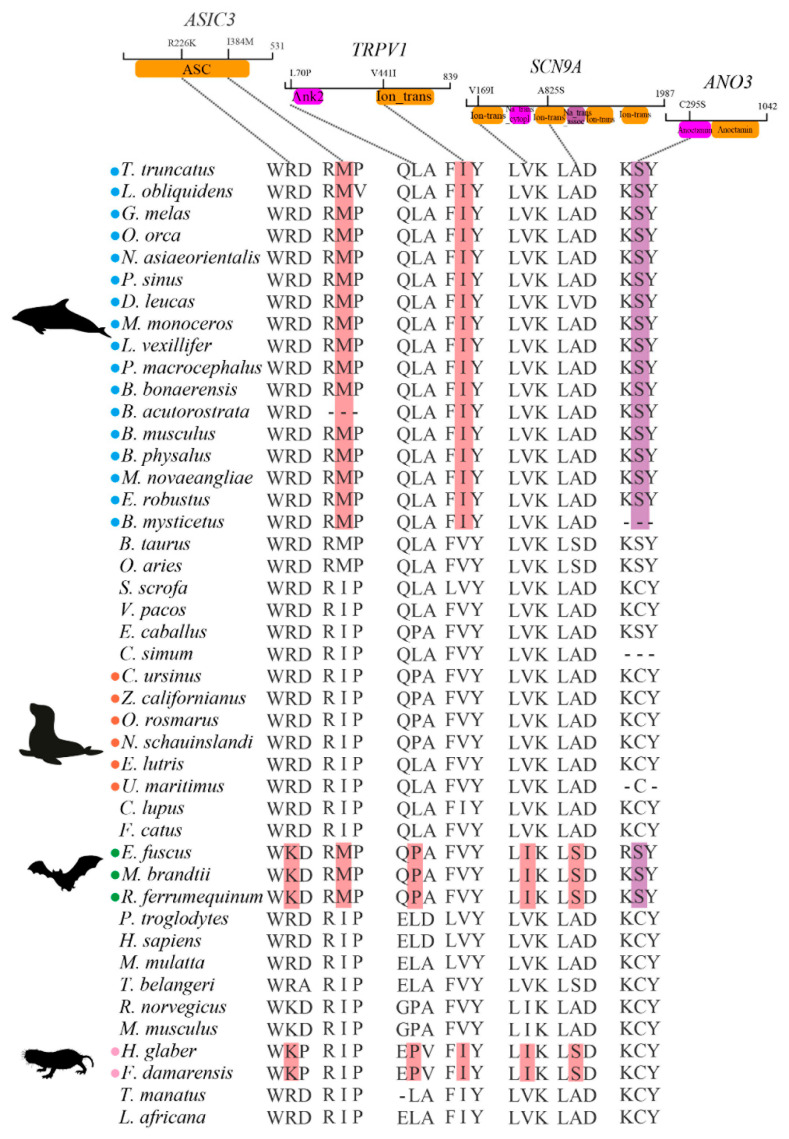
Potential convergent amino acids in species adapted to pain. Four groups of mammals adapted to pain are shown with circles of different colors: cetaceans (blue), other marine mammals (orange), bats (green), naked mole rats (pink). Amino acids in pink and purple are convergent amino acids associated with pain sensation and analgesia genes, respectively. These convergent amino acids are annotated on the protein domains (**on the top**). Amino acid positions are at the human scale.

We divided cetaceans, other marine mammals, bats, and naked mole rats into four groups of mammals that may be adapted to pain. Seven potential convergent amino acids in four genes among them were identified, when compared with a control set of housekeeping genes (no such molecular convergence). ASIC3 has two convergent amino acids, R226K of bats and naked mole rats, and I384M of cetaceans and bats; L70P of bats and naked mole rats, and V441I of cetaceans and naked mole rats were found in TRPV1; SCN9A has two convergent amino acids, V169I and A825S of bats and naked mole rats; C295S of cetaceans and bats was found in ANO3. Among them, ASIC3, *TRPV1*, and *SCN9A* are pain sensation genes and *ANO3* is an analgesia gene (Figure 4). 

### 3.5. Cetaceans-Specific Amino Acid Substitutions

**Figure 5:**
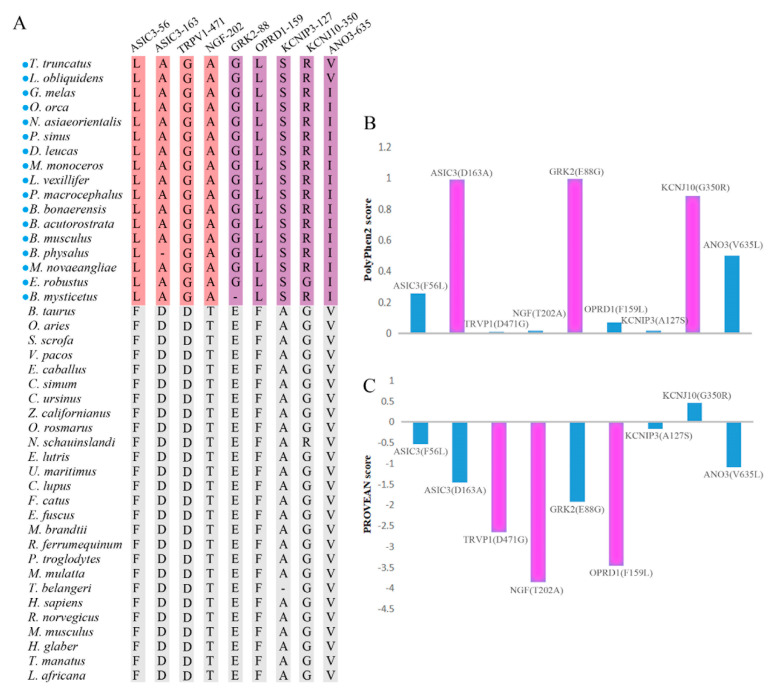
Cetaceans-specific amino acid substitutions. (**A**) Cetacean-specific amino acid substitutions identified in pain-related genes. Blue circles represent cetacean species. Amino acids in pink and purple are cetacean-specific amino acid substitutions identified in pain perception and analgesic genes, respectively. Amino acid positions are at the human scale. (**B**) Bar graph of the score of Polyphen2 predicting the effect of cetacean-specific amino acids on protein function. The purplish red bar graph shows that the scores are more than the threshold of 0.85, meaning that the protein’s function may be affected. (**C**) Bar graph of the PROVEAN score predicting the effect of cetacean-specific amino acids on protein function. The purplish red bar graph shows that the scores are less than the threshold of −2.5, meaning that the protein’s function may be affected.

We also found cetacean-specific amino acid substitutions, when compared with a control set of housekeeping genes (no such cetaceans-specific amino acid substitutions). Nine cetacean-specific amino acid substitutions were identified, of which ASIC3 F56L, ASIC3 D163A, TRPV1 D471G, and NGF T202A were detected in pain perception genes, and GRK2 E88G, OPRD1 F159L, KCNIP3 A127S, KCNJ10 G350R, and ANO3 V635I were detected in analgesic genes (Figure 5A). Furthermore, ASIC3 D163A, GRK2 E88G, and KCNJ10 G350R were predicted by Polyphen2 (scores > threshold of 0.85) to potentially affect protein function (Figure 5B, Supplementary Table S7); TRPV1 D471G, NGF T202A, and OPRD1 F159L were predicted by PROVEAN (scores < threshold of −2.5) to potentially affect protein function (Figure 5C, Supplementary Table S7).

## 4. Discussion

Pain is currently one of the most common disease symptoms in mammals. In 2018, the International Association for the Study of Pain (IASP) defined pain as “an unpleasant sensory and emotional experience associated or similarly associated with tissue damage or potential tissue damage.” From a neurobiological point of view, pain can be divided into three categories: nociceptive pain caused by noxious stimuli, inflammatory pain associated with tissue damage and immune cell infiltration, and pathological pain due to damage to the nervous system or its dysfunction [59]. Lack of nociceptive pain is a problem. There are reports on its molecular genetic cause—e.g., congenital insensitivity or indifference to pain due to in-frame deletions or rare missense of SCN9A, which encodes the Nav1.7, a voltage-gated sodium channel [60]. Another example is that mutations in the loss of function of nerve growth factor and the neurotrophic tyrosine kinase receptor TrkA typically lead to self-mutilation, bone fractures, multiple scars, joint deformities, amputations, and early death [61]. 

In this study, we discovered a pseudogenization of the acid-sensitive ion channel 4 gene (*ASIC4*) in all toothed whales except sperm whales. Furthermore, we showed that *ASIC4* experiences relaxed selection pressure on the branch on which we detected pseudogenization via selection pressure analysis. Notably, *ASIC4* is the newest member of the *ASIC* gene family of neuronal proton-gated cation channels. *ASIC4* itself is inactive and cannot form proton-gated ion channel complexes [62]. To some extent, it can promote the degradation of *ASIC1* or inhibits its expression [63]. *ASIC1* is the most abundant proton-gated ion channel gene in mammals. It is involved in the transmission of pain signals, and its degradation or reduced expression can lead to insensitivity to pain [64]. Therefore, the relaxed selection of *ASIC4* in toothed whales may have deprived it of its inhibitory effect on *ASIC1*, thereby making toothed whales more sensitive to pain. While examining the sperm whale and baleen whale lineages, we detected positive selection sites in important functional domains of the pain protein (i.e., ASIC3, ANO1, CCK, and SCN9A), also suggesting that sperm and baleen whales may also be more sensitive to pain. That is to say, different cetaceans may have evolved increased pain sensitivity through different paths, which is consistent with reports that bottlenose dolphins respond to painful stimuli through protest/withdrawal movements after acupuncture [19]. We also detected positive selection in pain sensation genes of species such as bats, naked mole rats, cows, and mice, which may be related to selective pain insensitivity. For example, primary afferent nociceptors in naked mole-rats are insensitive to acid stimuli, but nociceptors of them respond vigorously to capsaicin, and capsaicin-sensitive acid transduction by sensory neurons (TRPV1) is expressed in sensory neurons [11]. 

In addition, we specifically detected positive selection in three analgesic genes (*ARRB2*, *KCNK4* and *OPRL1*) in cetaceans. *ARRB2* is a member of the arrestin family. Its N-terminal domain can bind to phosphorylated GPCRs to form trimers and block pain signaling, exerting analgesic effects [65]. Thus, the 118th positive selection site (located in the N-terminal domain) of *ARRB2* in cetaceans may strengthen the negative regulation of pain signaling by *ARRB2*, thereby enhancing analgesia after suffering pain. It seems to explain that bottlenose dolphins show apparent indifference to pain after soft tissue injury [19].

Convergent evolution has always been a hot spot in the field of evolutionary biology. We identified seven potential convergent amino acids in four genes of species adapted to pain; most of these convergent amino acids were located in important functional domains of proteins, suggesting that different species that are adapted to pain may have undergone convergent evolution at the molecular level. Among the four genes, *SCN9A*-mapped mutations in pain disorders have been studied fully—e.g., p.Arg 222 His, evaluated from patients from a large family with early-onset pain symptoms, a familial mutation (I136V) and two sporadic mutations (I848T, V1316A) identified in patients with primary erythromelalgia (PE) [66,67,68]. The convergent amino acid substitutions (V169I and A825S) identified in this study, similar to these mutations, are located in the DI/S1 and DII important domains, respectively. Thus, these two convergent sites likely affect the function of *SCN9A* and contribute to pain insensitivity in bats and naked mole rats. Finally, we also found nine cetacean-specific amino acid substitutions and six of them were identified to affect the function of proteins. This means that cetaceans may have experienced a unique evolutionary trajectory different from other species adapted to pain. 

## 5. Conclusions

Overall, the above evidence reveals that in response to seawater environmental conditions, cetaceans might have undergone adaptive molecular evolution in pain and analgesic traits through different evolutionary patterns. On the one hand, cetaceans may be more sensitive to pain in certain situations, which is conducive to perceiving danger and avoiding risks, and on the other hand, they may be more effective in analgesia, which is beneficial for them to relieve pain after suffering “pain”.

## Figures and Tables

**Table 1:** Analysis of selective pressure relaxation in the *ASIC4* gene.

Models	ω	−lnL	Models Compared	2Δ(lnL)	*p* Value
One ratio ω(A)	0.087	−11,012.355			
One ratio ω = 1(B)	1.000	−12,064.363	B vs. A	2104.017	0
The branches with pseudogenized *ASIC4* have ω_2_, others ω_1_€	ω_1_ = 0.084 ω_2_ = 0.200	−11,008.339	A vs. C	8.031	0.004
The branches with pseudogenized *ASIC4* have ω_2_ = 1, others ω_1_(D)	ω_1_ = 0.084 ω_2_ = 1.000	−11,022.971	D vs. C	29.265	6.31221 × 10^−8^
Each branch has its own €(E)		−10,906.493	C vs. E	203.692	6.15578 × 10^−12^

0: The *p*-value approaches 0, but is displayed as 0 because the value is too small.

## Data Availability

Most of the data supporting the findings of this study can be found within the supplementary files, some other data are available from the corresponding author upon reasonable request.

## Supplementary Materials

The following are available online at https://www.mdpi.com/article/10.3390/ani12243571/s1, Table S1: 31 pain-related genes; Table S2. Information of genes sequences in this study; Table S3. Information of genome with Blast; Table S4. Primer sequence information; Table S5. Branch detected positive selection for pain-related genes by the branch model; Table S6. Branch detected positive selection for pain-related genes by the branch site model; Table S7. Predicted deleterious score by PROVEAN and PolyPhen2; Figure S1. Identification of ASIC4 gene of *L.vexillifer* and *N. asiaeorientalis*. The purplish red box indicates the stop codon.
