# Supplementary material for: Rubbing Salt in the Wound: Molecular Evolutionary Analysis of Pain-Related Genes Reveals the Pain Adaptation of Cetaceans in Seawater
Source: Animals (Basel). 2022 Dec 16;12(24):3571. doi: 10.3390/ani12243571 (PMC9774174; doi:10.3390/ani12243571)
Supplement: Supplementary file 1 [file animals-12-03571-s001.zip › animals-2020715-supplementary.pdf]

Table S1. 31 pain-related genes

Name	Effect (s)	Reference (s)
SCN9A	Conditional KO (cKO) of Scn9a in specific cell populations were shown to decrease sensitivity to various pain stimuli	[1]
SCN10A	The Possum mutant mice bearing a dominant hypermorphic mutation in Scn10a revealed higher sensitivity to noxious stimuli.	[1]
PRDM12	A key transcription factor in nociceptor neurogenesis.	[2]
ANO1	Are critical for sensory signal transduction	[3]
CCK	Pain modulation	[4]
MAPK1	A crucial role in the transduction of pain signal and contributes to pain hypersensitivity	[5]
MAPK3	The phosphorylation of MARK in the dorsal root ganglion (DRG) and dorsal horn neurons occurs in response to noxious stimulation of the peripheral tissue or electrical stimulation to the peripheral nerve	[6]
RUNX1	Runx1 plays essential roles during the development of sensory neurons involved in circuits mediating pain, itch, thermal sensation and sense of relative position	[7]
NGF	Mediating pain transmission and perception during adulthood	[8]
NTRK1	Autophosphorylates in response to NGF, thereby activating various pathways of intracellular pain signaling	[9]
CACNA1G	Cacna1g-null mice show attenuated neuropathic pain	[10]
TRPV1	Heat hyperalgesia, can be induced when TRPV1 is absent	[11]
TRPV4	As molecular sensors of chemical, thermal, and mechanical noxious stimuli to evoke the pain	[12]
P2RX3	P2X3 receptor involvement in pain states	[13]
P2RX4	P2X4R is dependent on ATP ligand-gated ion channel receptor, which can be activated by ATP and plays an important role in the information transmission of nerve system and the formation of pain	[14]
ASIC1	Activation of ASICs (Acid-sensing Ion Channels) leads physiologically to pain perception	[15]
ASIC3	The ASIC1 and ASIC3 isoforms are particularly important in sensory neurons	[16]
ASIC4	Acid-sensing ion channel (ASIC) 4 promotes the	[17]

	degradation of *ASIC1* or inhibits its expression.	
CA8	Impact on thermal antinociception	[18]
GRK2	Balancing GRK2 and EPAC1 levels prevents and relieves chronic pain	[19]
ARRB2	Association of polymorphisms in ARRB2 and clinical response to methadone for pain in advanced cancer	[20]
KCNA1	KCNA1 mutation in an Italian proband presenting with myokymia aggravated by painful contractures	[21]
KCNA2	KCNA2 can ameliorates neuropathic pain	[22]
TRAAK	TRAAK channels control pain produced by mechanical stimulation and both heat and cold pain perception in mice.	[23]
KCNIP3	Global gene knockout of *Kcnip3* enhances pain sensitivity	[24]
KCNJ10	Possible contributors to development of trigeminal neuralgia	[25]
ANO3	Painful-diabetic neuropathy patients show potentially pathogenic variants in *ANO3*	[26]
OPRM1	Association with morphine pain relief	[27]
OPRL1	Opioid-related nociceptin receptor gene	[28]
OPRK1	Associated with opioid effects	[29]
OPRD1	Impact on Morphine Analgesia	[30]

Table S2. Information of genes sequences in this study

Classification	Species name	Accession number							
		SCN9A	SCN10A	PRDM12	ANO1	CCK	MAPK1	MAPK3	RUNX1
	Tursiops truncatus	XM_019923191.2	XM_033864702.1	XM_033859290.1	XM_033861348.1	XM_019946658.2	XM_033837357.1	XM_033840604.1	XM_033856037.1
	Lagenorhynchus obliquidens	XM_027129270.1	XM_027097140.1	XM_027105972.1	XM_027090101.1	XM_027097155.1	XM_027088826.1	XM_027126453.1	XM_027100709.1
	Globicephala melas	XM_030852545.1	XM_030846194.1	XM_030838114.1	XM_030833236.1	XM_030846213.1	XM_030836288.1	XM_030871686.1	XM_030880838.1
	Orcinus orca	XM_004267254.3	XM_033401954.1	XM_004269370.1	XM_033402485.1	XM_004277868.2	XM_012535310.2	BLAST	XM_004264541.3
	Neophocaena asiaeorientalis	XM_024735947.1	XM_024768938.1	XM_024762220.1	XM_024749273.1	XM_024754161.1	XM_024763815.1	XM_024744495.1	XM_024758385.1
	Phocoena sinus	XM_032637962.1	XM_032648888.1	XM_032636300.1	XM_032638810.1	XM_032649894.1	XM_032602827.1	XM_032606377.1	XM_032630113.1
	Delphinapterus leucas	XM_022569944.1	XM_022563630.1	XM_022577993.1	XM_022591144.1	XM_022563667.1	XM_022551403.1	XM_022588367.2	XM_022552943.1
	Monodon monoceros	XM_029225298.1	XM_029244012.1	XM_029203485.1	XM_029208737.1	XM_029244108.1	XM_029234034.1	XM_029221610.1	XM_029214051.1
	Lipotes vexillifer	XM_007450046.1	XM_007468152.1	XM_007453140.1	XM_007466015.1	XM_007463234.1	XM_007451015.1	XM_007452780.1	XM_007446482.
	Physeter catodon	XM_024122208.2	XM_007110498.2	XM_024126094.1	XM_024133135.2	XM_007111562.2	XM_024120474.2	XM_024123717.1	XM_007127982.3
Cetartiodactyla	*Balaenoptera bonaerensis*	BLAST	BLAST	BLAST	BLAST	BLAST	BLAST	BLAST	BLAST
	Balaenoptera acutorostrata	XM_007183216.1	XM_007191455.1	XM_007194777.1	XM_007171269.1	XM_007193434.2	XM_007196295.2	XM_007186363.2	XM_007183740.2
	Balaenoptera musculus	XM_036859011.1	XM_036868976.1	XM_036856755.1	XM_036862640.1	XM_036869704.1	XM_036874241.1	XM_036824427.1	XM_036850892.1
	Balaenoptera physalus	BLAST	BLAST	BLAST	BLAST	BLAST	BLAST	BLAST	BLAST
	Megaptera novaeangliae	BLAST	BLAST	BLAST	BLAST	BLAST	BLAST	BLAST	BLAST
	Eschrichtius robustus	BLAST	BLAST	BLAST	BLAST	BLAST	BLAST	BLAST	BLAST
	Balaena mysticetus	BLAST	BLAST	BLAST	BLAST	BLAST	BLAST	BLAST	BLAST
	Bos taurus	XM_005202453.4	XM_015459489.2	NM_001206295.1	XM_024987196.1	XM_024983047.1	NM_175793.2	XM_005224919.4	XM_015473952.2
	Ovis aries	XM_027964893.1	XM_015102254.2	XM_027968320.1	XM_027959872.1	XM_027958055.1	XM_027956867.1	XM_015104158.2	XM_027961500.1
	Sus scrofa	XM_021076176.1	XM_021071677.1	XM_003122237.3	XM_021082670.1	NM_214237.2	NM_001198922.1	XM_021088019.1	XM_021068411.1
	Vicugna pacos	BLAST	XM_015236214.2	XM_031677214.1	XM_031691026.1	XM_015236328.1	XM_006213306.3	XM_006201256.2	XM_031688324.1
Perissodactyla	*Equus caballus*	XM_023623076.1	XM_014731551.2	XM_023629583.1	XM_023654624.1	XM_001501653.4	XM_023646801.1	XM_023616039.1	XM_023630093.1
	Ceratotherium simum	XM_004428233.2	XM_014790672.1	XM_025888525.1	XM_014795811.1	XM_004419556.2	XM_014796714.1	XM_004439522.1	XM_014786827.1

Carnivora	*Callorhinus ursinus*	BLAST	XM_025870888.1	XM_025888525.1	XM_025879019.1	XM_025870768.1	XM_025857341.1	BLAST	XM_025871737.1
	Zalophus californianus	XM_027590855.1	XM_027584950.1	XM_027616997.1	XM_027580751.1	XM_027585374.1	NA	XM_027613193.2	XM_027583260.1
	Odobenus rosmarus	XM_004396924.2	XM_004412301.1	XM_004392631.1	XM_004393795.1	XM_004414020.2	XM_004400515.2	XM_004397172.1	XM_012565636.1
	Neomonachus schauinslandi	XM_021703139.1	XM_021705864.1	XM_021691557.1	XM_021685595.1	XM_021678857.1	XM_021687870.1	XM_021700441.1	XM_021678047.1
	Enhydra lutris kenyoni	XM_022514231.1	XM_022521407.1	XM_022500350.1	XM_022507499.1	XM_022521534.1	XM_022496056.1	XM_022526505.1	XM_022516231.1
	Ursu maritimus	XM_008687054.1	XM_008710144.1	XM_008698873.1	XM_008699709.1	XM_008709882.1	XM_008697611.1	XM_008685240.1	XM_008701953.1
	Canis lupus familiaris	XM_022414839.1	NM_001003203.1	XM_025475499.2	XM_849396.5	XM_846822.5	NM_001110800.1	NM_001252035.1	XM_022413373.1
	Felis catus	XM_019838208.2	XM_023260720.1	XM_023242854.1	XM_023238951.1	XM_003992210.5	XM_003994973.4	XM_023247125.1	XM_023238765.1
Chiroptera	*Eptesicus fuscus*	XM_028140516.1	XM_028131986.1	XM_028127132.1	XM_028153731.1	XM_008154648.2	XM_008142643.2	XM_008152868.1	XM_028151671.1
	Myotis brandtii	XM_014546972.1	XM_014534926.1	BLAST	BLAST	XM_005860435.1	XM_014547095.1	XM_005881979.2	XM_014535203.1
	Rhinolophus ferrumequinum	XM_033112803.1	XM_033133226.1	XM_033123475.1	XM_033121009.1	XM_033132670.1	XM_033097341.1	NA	XM_033127961.1
Primates	*Pan troglodytes_*	XM_016949458.2	XM_009445190.2	XM_528445.5	XM_016921437.2	XM_009445273.3	XM_003317123.5	XM_016928783.2	XM_016938506.2
	Homo sapiens	NM_001365536.1	NM_006514.3	NM_021619.3	NM_018043.6	NM_000729.6	NM_002745.5	NM_002746.3	NM_001754.5
	Macaca mulatta	XM_015110280.2	XM_015131314.2	XM_015116553.2	NM_178642.6	XM_002802837.3	XM_015149801.2	XM_028840860.1	NM_001265962.1
Scandentia	*Tupaia chinensis*	BLAST	XM_009445190.2	XM_006164353.1	XM_014585285.2	XM_006155393.2	XM_014586841.2	XM_027771739.1	BLAST
Rodentia	*Rattus norvegicus*	NM_133289.1	NM_017247.1	XM_002726082.5	NM_001107564.1	NM_012829.2	NM_053842.2	NM_017347.2	XM_017598053.1
	Mus musculus	NM_001290674.1	NM_001205321.1	NM_001123362.1	XM_015113459.2	NM_001284508.2	NM_011949.3	NM_011952.2	NM_001111023.2
	Heterocephalus glaber	XM_021238552.1	XM_021259819.1	XM_004849106.2	XM_013074944.2	XM_004835106.3	XM_004843322.2	XM_004856274.2	XM_021256212.1
Sirenia	*Trichechus manatus*	NA	XM_023732211.1	XM_004375840.1	XM_012557644.1	XM_004368424.1	XM_023740543.1	XM_004386624.3	XM_023741332.1
Proboscidea	*Loxodonta africana*	XM_010602854.2	XM_023554824.1	XM_003407564.1	XM_023541837.1	XM_003409718.1	XM_010598893.2	XM_010598495.2	XM_023548425.1
Classification	Species name	Accession number							
		NGF	NTRK1	CACNA1G	TRPV1	TRPV4	P2RX3	P2RX4	ASIC1
Cetartiodactyla	*Tursiops truncatus*	XM_004318079.3	XM_033857030.1	XM_033847346.1	XM_019926881.1	XM_033837066.1	XM_033860719.1	XM_004330179.3	XM_033866740.1
	Lagenorhynchus obliquidens	XM_027090969.1	XM_027095266.1	XM_027094399.1	XM_027130680.1	XM_027124505.1	XM_027114691.1	XM_027124824.1	XM_027106231.1
	Globicephala melas	XM_030869133.1	XM_030842062.1	XM_030839818.1	XM_030861763.1	XM_030860606.1	XM_030864399.1	XM_030860287.1	XM_030835108.1
	Orcinus orca	XM_004263233.2	XM_004284532.2	XM_004282573.2	XM_004267034.2	XM_033408605.1	XM_004264119.3	XM_004276721.3	XM_004274347.2
	Neophocaena asiaeorientalis	XM_024764943.1	XM_024748205.1	XM_024743746.1	XM_024736548.1	XM_024741485.1	XM_024749217.1	XM_024758552.1	XM_024736891.1

	Phocoena sinus	XM_032633146.1	XM_032637667.1	XM_032615915.1	XM_032616759.1	XM_032602893.1	XM_032640910.1	XM_032653862.1	XM_032645742.1
	Delphinapterus leucas	XM_030760001.1	XM_022559547.2	XM_022557857.2	XM_022595823.2	XM_030764334.1	XM_022591980.1	XM_022597833.1	XM_022572201.2
	Monodon monoceros	XM_029230947.1	XM_029230923.1	XM_029211329.1	XM_029211930.1	XM_029228313.1	XM_029208585.1	XM_029216388.1	XM_029205422.1
	Lipotes vexillifer	XM_007454787.1	XM_007457870.1	XM_007465006.1	XM_007454184.1	XM_007448216.1	XM_007455714.1	XM_007448718.1	XM_007452281.1
	Physeter catodon	XM_007103024.2	XM_024748205.1	XM_024130076.1	XM_007125466.3	XM_028480246.1	XM_007115874.2	XM_007123245.3	Orthemam
	Balaenoptera bonaerensis	BLAST	BLAST	BLAST	BLAST	BLAST	BLAST	BLAST	BLAST
	Balaenoptera acutorostrata	XM_007169341.1	XM_007172018.1	XM_007176453.1	XM_022595823.2	XM_007180110.1	XM_007196450.1	XM_007189529.1	XM_007179337.1
	Balaenoptera musculus	XM_036840585.1	XM_036836292.1	XM_036837223.1	XM_036838427.1	BLAST	XM_036861777.1	XM_036875009.1	XM 036864743.1
	Balaenoptera physalus	BLAST	BLAST	BLAST	BLAST	BLAST	BLAST	BLAST	BLAST
	Megaptera novaeangliae	BLAST	BLAST	BLAST	BLAST	BLAST	BLAST	BLAST	BLAST
	Eschrichtius robustus	BLAST	BLAST	BLAST	BLAST	BLAST	BLAST	BLAST	BLAST
	Balaena mysticetus	BLAST	BLAST	BLAST	BLAST	BLAST	BLAST	BLAST	BLAST
	Bos taurus	NM_001099362.1	XM_002685966.6	NM_001193140.3	XM_015458647.2	NM_001192385.3	XM_024975926.1	NM_001034049.1	NM_001192933.1
	Ovis aries	XM_004002369.4	XM_027976575.1	XM_027974720.1	XM_027975474.1	XM_027956636.1	XM_015101165.2	XM_004017580.4	XM_004006368.4
	Sus scrofa	XM_021089997.1	XM_001929525.5	XM_021067168.1	XM_013981216.2	NM_001130729.1	XM_003353865.5	XM_021073038.1	XM_021091639.1
	Vicugna pacos	XM_006213697.3	XM_015248158.2	XM_031676276.1	XM_006213341.2	XM_031669982.1	XM_006214057.2	XM_015239748.2	XM_006202956.2
Perissodactyla	*Equus caballus*	XM_001496187.5	XM_023640974.1	XM_023652720.1	XM_014727972.2	XM_023648063.1	XM_001504914.5	XM_023647186.1	XM_023643524.1
	Ceratotherium simum	XM_004420625.2	XM_004436243.2	XM_004434702.2	XM_004433273.2	XM_004429881.2	XM_004437362.2	XM_004429997.2	XM_004429001.2
Carnivora	*Callorhinus ursinus*	XM_025872630.1	XM_027774484.1	XM_025859233.1	XM_025851492.1	XM_025889376.1	XM_025879400.1	XM_025889775.1	XM_004429001.2
	Zalophus californianus	XM_027618984.1	XM_027612047.2	XM_027625233.2	XM_027567722.2	XM_027577421.1	XM_027579308.2	XM_027576877.2	XM_027592606.1
	Odobenus rosmarus	XM_004401273.2	XM_004402890.1	XM_004395166.1	XM_004412121.2	XM_004416178.2	XM_004409265.2	XM_004395865.1	XM_004400839.1
	Neomonachus schauinslandi	XM_021690213.1	XM_021681954.1	XM_021703916.1	XM_021704294.1	XM_021703490.1	XM_021684842.1	XM_021698605.1	XM_021704848.1
	Enhydra lutris kenyoni	BLAST	XM_022490805.1	XM_022509041.1	XM_022521029.1	XM_022517275.1	XM_022507296.1	XM_022517426.1	XM_022519560.1
	Ursu maritimus	XM_008697772.1	BLAST	XM_008688551.1	XM_018064037.1	XM_008707622.1	XM_008694134.1	XM_008688985.1	XM_008704561.1
	Canis lupus familiaris	NM_001194950.1	XM_022421240.1	XM_022423104.1	NM_001003970.1	NM_001127315.1	XM_038424307.1	XM_038436608.1	XM_022411216.1
	Felis catus	XM_004001117.4	XM_023247543.1	XM_023244164.1	XM_023244720.1	XM_023241517.1	XM_019812424.1	XM_023241408.1	XM_019834899.2
Chiroptera	*Eptesicus fuscus*	XM_028154129.1	XM_008155630.1	XM_028128553.1	XM_008156768.2	XM_008158970.2	XM_008159313.2	BLAST	XM_028144343.1
	Myotis brandtii	XM_005859963.2	BLAST	XM_005868217.2	XM_005869032.2	XM_014537663.1	XM_005885097.2	XM_014547038.1	XM_005877303.2

	Rhinolophus ferrumequinum	XM_033093056.1	XM_033091995.1	XM_033089393.1	XM_033089961.1	XM_033098558.1	XM_033121437.1	XM_033097404.1	XM_033116516.1
Primates	*Pan troglodytes*	NM_001012437.1	XM_009434644.3	XM_016932515.2	BLAST	XM_016924151.2	XM_016920882.2	XM_016924411.2	XM_009425433.3
	Homo sapiens	NM_002506.3	NM_002529.4	NM_018896.5	NM_080704.4	XM_011538630.2	XM_011545069.2	NM_001256796.2	NM_020039.4
	Macaca mulatta	XM_015148902.2	XM_001114243.4	XM_015119270.2	XM_028835887.1	XM_028829845.1	XM_001103269.4	XM_015152983.2	NM_001261065.1
Scandentia	*Tupaia chinensis*	XM_006149649.2	XM_027774484.1	XM_027773865.1	XM_006167590.3	XM_016924151.2	XM_006160805.1	XM_014588118.1	XM_006168179.2
Rodentia	*Rattus norvegicus*	NM_001277055.1	NM_021589.1	NM_001308302.1	NM_031982.1	NM_023970.1	NM_031075.2	NM_031594.2	NM_024154.2
	Mus musculus	NM_013609.3	NM_001033124.1	XM_011248686.3	NM_001001445.2	NM_022017.3	XM_030250183.2	XM_006530196.4	NM_001289791.2
	Heterocephalus glaber	XM_021265739.1	XM_004871248.2	XM_021239950.1	NM_001279859.1	XM_004843606.2	XM_021248323.1	XM_004843834.3	NM_001279840.1
Sirenia	*Trichechus manatus*	XM_004380362.2	XM_023742603.1	XM_023732605.1	XM_004376247.2	XM_012556273.1	XM_004389182.2	BLAST	XM_004388310.1
Proboscidea	*Loxodonta africana*	XM_010589594.2	XM_003415164.1	XM_003414620.2	XM_023556293.1	XM_023559499.1	XM_003421330.3	XM_023539281.1	XM_023539332.1
Classification	Species name	Accession number							
		ASIC3	NRF2	CA8	GRK2	ARRB2	KCNA1	KCNA2	TRAAK
Cetartiodactyla	*Tursiops truncatus*	XM_033863484.1	XM_033859985.1	XM_033843081.1	XM_033861227.1	XM_033847483.1	XM_004323632.1	XM_004317089.3	XM_033861680.1
	Lagenorhynchus obliquidens	XM_027101505.1	XM_027127372.1	XM_027128036.1	XM_027090661.1	XM_027115153.1	XM_027093349.1	XM_027096237.1	XM_027090325.1
	Globicephala melas	XM_030854219.1	XM_030851115.1	XM_030859688.1	XM_030833385.1	XM_030862260.1	XM_030834445.1	XM_030868983.1	XM_030833091.1
	Orcinus orca	XM_004281188.2	XM_033423631.1	XM_004274987.3	XM_004277999.3	BLAST	XM_004279022.2	XM_033435001.1	XM_004264293.3
	Neophocaena asiaeorientalis	XM_024754494.1	XM_024732874.1	XM_024742663.1	XM_024748786.1	XM_024735742.1	XM_024733309.1	XM_024742066.1	XM_024749412.1
	Phocoena sinus	XM_032642543.1	XM_032638174.1	XM_032610210.1	XM_032640172.1	XM_032617324.1	XM_032646038.1	XM_032635726.1	XM_032641081.1
	Delphinapterus leucas	XM_022577735.2	XM_022570099.2	XM_022595338.2	XM_022591301.1	XM_030763303.1	XM_022577354.1	XM_022584109.1	XM_022591622.2
	Monodon monoceros	XM_029233835.1	XM_029225511.1	XM_029222668.1	XM_029207913.1	XM_029211189.1	XM_029205951.1	XM_029229097.1	XM_029208991.1
	Lipotes vexillifer	XM_007469835.1	XM_007451096.1	XM_007471469.1	XM_007450442.1	XM_007457720.1	XM_007469438.1	XM_007449287.1	XM_007462067.1
	Physeter catodon	XM_028489455.1	XM_007105356.3	XM_024127047.1	XM_024133396.2	XM_028498954.1	XM_024129221.1	XM_028489025.1	XM_007102613.3
	Balaenoptera bonaerensis	BLAST	BLAST	BLAST	BLAST	BLAST	BLAST	BLAST	BLAST
	Balaenoptera acutorostrata	BLAST	XM_007190417.2	XM_007168573.1	XM_007171049.1	XM_028168194.1	XM_007170198.2	XM_007169434.1	XM_007174467.1
	Balaenoptera musculus	XM_036864236.1	XM_036857224.1	XM_036830405.1	XM_036861662.1	XM_036837682.1	XM_036864598.1	XM_036830087.1	XM_036859318.1
	Balaenoptera physalus	BLAST	BLAST	BLAST	BLAST	BLAST	BLAST	BLAST	BLAST
	Megaptera novaeangliae	BLAST	BLAST	BLAST	BLAST	BLAST	BLAST	BLAST	BLAST
	Eschrichtius robustus	BLAST	BLAST	BLAST	BLAST	BLAST	BLAST	BLAST	BLAST

	Balaena mysticetus	BLAST	BLAST	BLAST	BLAST	BLAST	BLAST	BLAST	BLAST
	Bos taurus	NM_001191251.3	NM_001011678.2	NM_001083690.2	NM_174710.2	NM_001205277.2	XM_005207204.4	XM_005204159.4	XM_024987649.1
	Ovis aries	XM_027969009.1	XM_015093345.2	XM_027972975.1	XM_027959352.1	XM_027975066.1	XM_012176138.2	XM_004002309.3	XM_027959629.1
	Sus scrofa	XM_021078870.1	XM_005671981.3	XM_021089339.1	XM_021078541.1	XM_021067791.1	XM_021092437.1	XM_021088699.1	XM_013994155.2
	Vicugna pacos	XM_031673805.1	XM_031678320.1	XM_015239558.2	XM_006210695.3	XM_006217562.2	XM_015244955.2	XM_015244353.2	XM_031691105.1
Perissodactyla	*Equus caballus*	NM_001195636.1	XM_023622057.1	XM_001496473.6	XM_023654544.1	XM_023653377.1	NM_001256931.1	XM_023641436.1	XM_001489646.6
	Ceratotherium simum	XM_004430614.1	XM_004426661.2	XM_004435728.2	XM_004437694.2	XM_014789607.1	XM_004438743.2	XM_014780045.1	XM_004437551.2
	Callorhinus ursinus	XM_025862183.1	XM_025849627.1	XM_025870626.1	XM_025879353.1	XM_025864300.1	XM_025851967.1	XM_025872383.1	XM_025878368.1
	Zalophus californianus	XM_027574214.1	XM_027588598.1	XM_027619374.1	XM_027580423.2	XM_027625051.1	XM_027594581.1	XM_027621322.1	XM_027579850.1
	Odobenus rosmarus	XM_012565386.1	XM_004403725.2	XM_004398774.2	XM_004393851.2	XM_004398561.1	XM_004413371.2	XM_004407154.2	XM_004393996.1
Carnivora	*Neomonachus schauinslandi*	XM_021693043.1	XM_021703373.1	XM_021688351.1	XM_021685101.1	XM_021694560.1	XM_021690657.1	XM_021690143.1	XM_021684991.1
	Enhydra lutris kenyoni	XM_022513853.1	XM_022495499.1	XM_022515585.1	XM_022506534.1	XM_022524703.1	XM_022522394.1	XM_022496961.1	XM_022507299.1
	Ursu maritimus	BLAST	XM_008687171.1	XM_008706724.1	XM_008709169.1	XM_008688069.1	BLAST	XM_008704045.1	XM_008709114.1
	Canis lupus familiaris	XM_022403869.1	XM_038447161.1	XM_544094.6	XM_846992.4	XM_022418470.1	NM_001313824.1	NM_001003329.1	XM_005631548.3
	Felis catus	XM_019826020.1	XM_003990893.5	XM_023248778.1	XM_023240104.1	XM_023243688.1	XM_019834392.2	XM_019837595.2	XM_019812622.2
	Eptesicus fuscus	XM_028135579.1	XM_028140905.1	XM_008143311.2	XM_008146878.2	XM_028132982.1	XM_008143755.2	XM_028154036.1	XM_028135921.1
Chiroptera	*Myotis brandtii*	XM_005880096.2	XM_005857611.2	XM_005871667.2	XM_014538223.1	XM_005882584.1	XM_005877017.2	XM_014534613.1	XM_014538011.1
	Rhinolophus ferrumequinum	XM_033099212.1	XM_033111983.1	XM_033126672.1	XM_033120706.1	XM_033089486.1	XM_033117984.1	XM_033092490.1	XM_033119725.1
	*Pan troglodytes_*	XM_028846475.1	XM_001145876.5	XM_024345179.1	XM_508589.6	XM_016931274.1	XM_003313436.4	NM_001265976.1	NA
Primates	*Homo sapiens*	NM_020321.3	NM_006164.5	NM_004056.6	NM_001619.5	NM_001257328.2	NM_000217.3	NM_004974.4	NM_033310.3
	Macaca mulatta	XM_009454575.3	NM_001257607.1	NM_001261295.1	XM_028833177.1	XM_015118329.1	XM_001102114.4	XM_001162925.3	XM_016921158.2
Scandentia	*Tupaia chinensis*	XM_006150218.2	XM_027773121.1	NA	XM_027769428.1	XM_006151854.3	XM_006172022.3	XM_027772296.1	XM_006149842.3
	Rattus norvegicus	NM_173135.1	NM_031789.2	NM_001009662.1	NM_012776.1	NM_012911.1	NM_173095.3	NM_012970.3	XM_008760056.2
Rodentia	*Mus musculus*	NM_183000.2	NM_010902.4	NM_007592.3	NM_130863.2	NM_001271358.1	NM_010595.3	NM_008417.5	XM_006526720.3
	Heterocephalus glaber	XM_013078018.2	XM_004857736.2	XM_004842145.2	XM_004852257.3	XM_004857272.3	XM_004869373.3	XM_004853692.2	XM_021264669.1
Sirenia	*Trichechus manatus*	XM_004372736.1	XM_023730537.1	XM_023727774.1	XM_023738164.1	XM_023731114.1	XM_004381168.2	XM_004390417.2	XM_004383782.2
Proboscidea	*Loxodonta africana*	XM_023548044.1	XM_003406202	XM_003408321.3	XM_023559549.1	XM_023556361.1	XM_003410634.3	XM_023547452.1	XM_003419447.3
Classification	Species name	Accession number							

		KCNIP3	KCNJ10	ANO3	OPRM1	OPRL1	OPRK1	OPRD1
Cetartiodactyla	*Tursiops truncates*	XM_033838401.1	XM_033856559.1	XM_019948278.2	XM_019951587.2	XM_033839461.1	XM_019925440.1	XM_033855246.1
	Lagenorhynchus obliquidens	XM_027128984.1	XM_027095083.1	XM_027115153.1	XM_027083030.1	XM_027086652.1	XM_027127872.1	XM_027109413.1
	Globicephala melas	XM_030838967.1	XM_030841942.1	XM_030864848.1	XM_030853121.1	XM_030841458.1	XM_030859828.1	XM_030865523.1
	Orcinus orca	XM_004277795.2	XM_004284454.1	XM_004263929.2	XM_012536769.1	XM_004282377.2	XM_004275020.2	XM_004266575.2
	Neophocaena asiaeorientalis	XM_024744018.1	XM_024750476.1	XM_024764654.1	XM_024748046.1	XM_024750256.1	XM_024742594.1	XM_024766814.1
	Phocoena sinus	XM_032653620.1	XM_032648098.1	XM_032640382.1	XM_032650842.1	XM_032604328.1	XM_032610055.1	XM_032642157.1
	Delphinapterus leucas	XM_022599027.1	XM_022559754.1	XM_022583740.2	XM_022559871.1	XM_022597651.2	XM_022556842.1	XM_022552068.2
	Monodon monoceros	XM_029232659.1	XM_029228840.1	XM_029209693.1	XM_029224170.1	XM_029222037.1	XM_029222825.1	XM_029229318.1
	Lipotes vexillifer	XM_007470197.1	XM_007461279.1	XM_007462608.1	XM_007458731.1	XM_007462220.1	XM_007464020.1	XM_007461817.1
	Physeter catodon	XM_007118470.1	XM_007129619.3	XM_024119081.1	XM_007129176.2	XM_024128409.1	XM_024127031.1	XM_024125424.1
	Balaenoptera bonaerensis	BLAST	BLAST	BLAST	BLAST	BLAST	BLAST	BLAST
	Balaenoptera acutorostrata	XM_007197357.2	XM_007171784.2	XM_007180984.2	XM_007186136.1	XM_007185174.1	XM_007168611.1	XM_007170618.2
	Balaenoptera musculus	XM_036874091.1	XM_036852607.1	XM_036861881.1	XM_036872213.1	XM_036825787.1	XM_036830487.1	XM_036867319.1
	Balaenoptera physalus	BLAST	BLAST	BLAST	BLAST	BLAST	BLAST	BLAST
	Megaptera novaeangliae	BLAST	BLAST	BLAST	BLAST	BLAST	BLAST	BLAST
	Eschrichtius robustus	BLAST	BLAST	BLAST	BLAST	BLAST	BLAST	BLAST
	Balaena mysticetus	BLAST	BLAST	BLAST	BLAST	BLAST	BLAST	BLAST
	Bos taurus	NM_001075612.2	NM_001081601.1	NM_001191315.2	NM_174408.2	XM_015474110.2	NM_001318764.1	NM_001191148.1
	Ovis aries	XM_027967108.1	XM_004002660.4	XM_027979573.1	XM_004011420.3	XM_027976897.1	XM_004011957.4	XM_027965410.1
	Sus scrofa	XM_021087059.1	XM_005663208.3	XM_013987284.2	NM_001001538.1	NM_214176.1	XM_003355059.5	XM_003356260.5
	Vicugna pacos	XM_031692051.1	XM_006215640.3	XM_031681078.1	XM_006197712.2	XM_015243904.2	XM_006204664.3	XM_006196882.2:
Perissodactyla	*Equus caballus*	XM_023618686.1	XM_001491211.5	XM_014741888.2	XM_014738201.2	XM_001495485.5	XM_023648620.1	XM_023634919.1
	Ceratotherium simum	XM_004435806.2	XM_004442773.2	XM_014783643.1	XM_014795480.1	XM_004430168.2	XM_004435697.2	XM_004425847.1
Carnivora	*Callorhinus ursinus*	XM_025867613.1	XM_025860752.1	XM_025879336.1	XM_025866359.1	XM_025865055.1	XM_025871427.1	XM_025856040.1
	Zalophus californianus	XM_027623931.2	XM_027613702.2	XM_027580367.1	XM_027601049.2	XM_027623227.1	XM_027599696.1	XM_027618330.2
	Odobenus rosmarus	XM_004399843.2	XM_004407858.2	XM_004416870.1	XM_012566802.1	XM_004410095.1	XM_004416041.2	XM_004394640.1
	Neomonachus schauinslandi	XM_021697498.1	XM_021681916.1	XM_021685869.1	XM_021693699.1	XM_021677749.1	XM_021688277.1	XM_021683630.1

	Enhydra lutris kenyoni	XM_022520465.1	XM_022490971.1	XM_022506952.1	XM_022520971.1	XM_022494199.1	XM_022515599.1	XM_022496667.1
	Ursu maritimus	XM_008700533.1	XM_008700702.1	XM_008686892.1	XM_008688566.1	XM_008688143.1	XM_008706685.1	XM_008694831.1
	Canis lupus familiaris	XM_003639596.4	XM_005640901.3	XM_534094.6	NM_001314122.1	XM_014107305.2	XM_544080.6	XM_544455.6
	Felis catus	XM_003984274.5	XM_019822326.2	XM_023239658.1	XM_019831412.1	XM_019826183.1	XM_023248439.1	XM_003989776.4
	Eptesicus fuscus	XM_008160573.2	XM_008154256.2	XM_028156716.1	XM_008154677.2	XM_008157153.2	XM_028147996.1	XM_008147978.2
Chiroptera	*Myotis brandtii*	XM_005860465.2	XM_005857772.2	XM_014545226.1	XM_014545053.1	XM_005885520.2	XM_005857314.2	XM_005875244.2
	Rhinolophus ferrumequinum	XM_033100946.1	XM_033093604.1	XM_033120577.1	XM_033094713.1	XM_033094198.1	XM_033125705.1	XM_033115868.1
	*Pan troglodytes_*	XM_016948932.2	XM_009435435.2	XM_001134720.6	NM_001033915.1	XM_016938297.2	XM_009455386.3	XM_513259.6
Primates	*Homo sapiens*	NM_013434.5	NM_002241.5	NM_001313726.1	XM_017010903.2	NM_001318853.2	NM_001318497.2	NM_000911.4
	Macaca mulatta	NM_001257864.1	XM_015110279.2	NM_001265904.1	NM_001032824.1	NM_001320615.1	NM_001321098.2	XM_001113252.4
Scandentia	*Tupaia chinensis*	NA	XM_006168841.3	XM_027775367.1	XM_006139989.3	XM_006167092.1	XM_006140344.2	BLAST
	Rattus norvegicus	NM_032462.2	XM_008769737.2	XM_017602571.1	XM_008758731.2	NM_001318947.1	XM_017593203.1	NM_012617.1
Rodentia	*Mus musculus*	NM_019789.4	XM_006496677.5	NM_001128103.2	XM_006512437.5	NM_001318919.1	NM_001318735.1	NM_013622.3
	Heterocephalus glaber	XM_004844345.3	XM_021239684.1	XM_004851573.3	NM_001267590.1	NM_001310286.1	NM_001310230.1	XM_004850778.2
Sirenia	*Trichechus manatus*	XM_004369351.1	XM_004390204.2	XM_023737820.1	XM_004368814.2	XM_004370148.1	XM_004375606.1	XM_004377022.1
Proboscidea	*Loxodonta africana*	XM_023541135.1	XM_003415179.3	XM_023550773.1	XM_010588919.2	XM_003421774.3	XM_003408315.3	XM_003415273.3

BLAST: obtained from the local database blast; NA: Not available

Table S3. Information of genome with Blast

Species	Genome
Tursiops truncatus	GCF_011762595.1_mTurTru1.mat.Y
Lagenorhynchus obliquidens	GCF_003676395.1_ASM367639v1
Globicephala melas	GCF_006547405.1_ASM654740v1
Orcinus orca	GCF_000331955.2 _Oorc_1.1
Neophocaena asiaeorientalis	GCF_003031525.1_Neophocaena_asiaeorientalis_V1
Phocoena sinus	GCF_008692025.1_mPhoSin1.pri
Delphinapterus leucas	GCF_002288925.2_
Monodon monoceros	GCF_005190385.1_NGI_Narwhal_1
Lipotes vexillifer	GCF_000442215.1_Lipotes_vexillifer_v1
Physeter catodon	GCF_002837175.2_ASM283717v2
Balaenoptera bonaerensis	GCA_000978805.1_ASM97880v1
Balaenoptera acutorostrata scammoni	GCF_000493695.1_BalAcu1.0
Balaenoptera musculus	GCF_009873245.2_mBalMus1.pri.v3
Balaenoptera physalus	GCA_023338255.1_SBiKF_Bphy_ph2
Megaptera novaeangliae	https://dataverse.harvard.edu/file.xhtml?persistentId=doi:10.7910/DVN/ADHX1O/0J3CYS&version=1.0
Eschrichtius robustus	GCA_002189225.1_ASM218922v1
Balaena mysticetus	http://www.bowhead-whale.org/annotations/
Bos taurus	GCF_002263795.1_GCA_002263795.2
Ovis aries	GCF_002742125.1_Oar_rambouillet_v1.0
Sus scrofa	GCF_000003025.6_Sscrofa11.1
Vicugna pacos	GCF_000164845.3_VicPac3.1
Equus caballus	GCF_002863925.1_EquCab3.0
Ceratotherium simum	GCF_000283155.1_CerSimSim1.0
Callorhinus ursinus	GCF_003265705.1_ASM326570v1
Zalophus californianus	GCF_900631625.1_mZalCal1.pri.v2
Odobenus rosmarus	GCF_000321225.1_Oros_1.0
Neomonachus schauinslandi	GCF_002201575.1_ASM220157v1
Enhydra lutris kenyoni	GCF_002288905.1_ASM228890v2
Ursu maritimus	GCF_000687225.1_UrsMar_1.0
Canis lupus familiaris	GCF_005444595.1_UMICH_Zoey_3.1
Felis catus	GCF_000181335.3_Felis_catus_9.0
Eptesicus fuscus	GCF_000308155.1_EptFus1.0

Myotis brandtii	GCA_000412655.1_ASM41265v1
Rhinolophus ferrumequinum	GCF_004115265.1_mRhiFer1_v1
*Pan troglodytes_*	GCF_002880755.1_Clint_PTRv2
Homo sapiens	GCF_000001405.39_GRCh38.p13
Macaca mulatta	GCF_000002255.3_Mmul_051212
Tupaia chinensis	GCF_000334495.1_TupChi_1.0
Rattus norvegicus	GCF_000002265.2_Rn_Celera
Mus musculus	GCF_000001635.27_GRCm39
Heterocephalus glaber	GCF_000247695.1_HetGla_female_1.0
Trichechus manatus	GCF_000243295.1_TriManLat1.0
Loxodonta africana	GCF_000001905.1_Loxafr3.0

Table S4. Primer sequence information

Primer name	Sequence（5'-3'）	Annealing temperature（℃）	Length of product（bp）
N. asiaeorientalis-F	TCTTCCCGTTTGCCCTGAGTT	61.94	548
N. asiaeorientalis-R	TCAGATTGGCCAGGTGGAAGA	61.12	548
L. vexillifer- F	TTCCCGTTTGCCCTGAGTTT	60.11	547
L. vexillifer- R	GTCAGATTGGCCAGGTGGAA	59.96	547

Table S5. Branch detected positive selection for pain-related genes by the branch model

**Gene**	**Branch**	**ω value**	**2ΔlnL**	p^{adjust} **value**
ASIC3	*E. robustus*	1.299	8.209316	$p<0.05$
TRPV4	*M. novaeanglise*	1.054	15.56283	$p<0.05$
CA8	LCA of *B. mysticetus &B. bonaerensis*	1.262	12.820818	$p<0.05$

LAC: last common ancestor

Table S6. Branch detected positive selection for pain-related genes by the branch site model

**Gene**	**Branch**	**ω value（ω0 ω1 ω2）**	**2ΔlnL**	**p^{adjust} value**	**Positively selected sites (PP > 0.8)**
SCN9A	LAC of of *Ovis aries* & *T. truncatus*	0.041 1.000 130.796	7.792844	*p*<0.05	127 L 0.941
	M. brandtii	0.040 1.000 12.300	9.616722	*p*<0.05	564 G 0.998**; 1140 A 0.998**;1493 Q 0.995**
SCN10A	*B. taurus*	0.059 1.000 11.250	8.231646	*p*<0.05	1866 L 0.981*
	*H. glabe*r	0.040 1.000 12.300	9.540488	*p*<0.05	1347 A 0.902
ANO1	*LAC* of *B. bonaerensis & B.acutorostrata*	0.027 1.000 142.571	9.665078	*p*<0.05	36 T 0.979*
	R. norvegicus	0.027 1.000 138.433	16.22956	*p*<0.05	473 K 0.996**
CCK	*P. catodon*	0.109 1.000 132.637	10.509472	*p*<0.05	37 E 0.997**;98 M 0.942; 112 E [illegible]
TRPV1	*H. glaber*	0.059 1.000 58.050	9.540488	*p*<0.05	1347 A 0.902
ASIC3	*B. physalus*	0.075 1.000 349.853	16.91663	*p*<0.05	468 D 0.998**
	M. mulatta	0.072 1.000 28.035	21.05658	*p*<0.05	357 I 0.964*;399 L 0.946
ARRB2	*B. physalus*	0.011 1.000 181.298	7.824406	*p*<0.05	124 G 0.984*
KCNK4	*E. robustus*	0.070 1.000 109.557	6.498806	*p*<0.05	47 L 0.930; 103 T 0.932; 104 I 0.999**
OPRL1	LAC *of Cetacea*	0.037 1.000 3.741	8.14323	*p*<0.05	11 E 0.957*;19 Q 0.982*; 195 D 1.000**;196 E 0.801; 225 I
OPRM1	*S. scrofa*	0.032 1.000 55.104	11.033892	*p*<0.05	47 I 0.993**

LAC: last common ancestor

Table S7. Predicted deleterious score by PROVEAN and PolyPhen2

Variant	Predicted deleterious score	
	PROVEAN	PolyPhen2
ASIC3(F56L)	-0.540	0.256
ASIC3(D163A)	-1.471	0.987
TRVP1(D471G)	-2.668	0.010
NGF(T202A)	-3.866	0.015
GRK2(E88G)	-1.924	0.992
OPRD1(F159L)	-3.467	0.069
KCNIP3(A127S)	-0.172	0.016
KCNJ10(G350R)	0.450	0.885
ANO3(V635L)	-1.106	0.498

Purplish red labeled values are scores for cetacean-specific amino acid substitutions predicted to affect protein function.

Figure S1

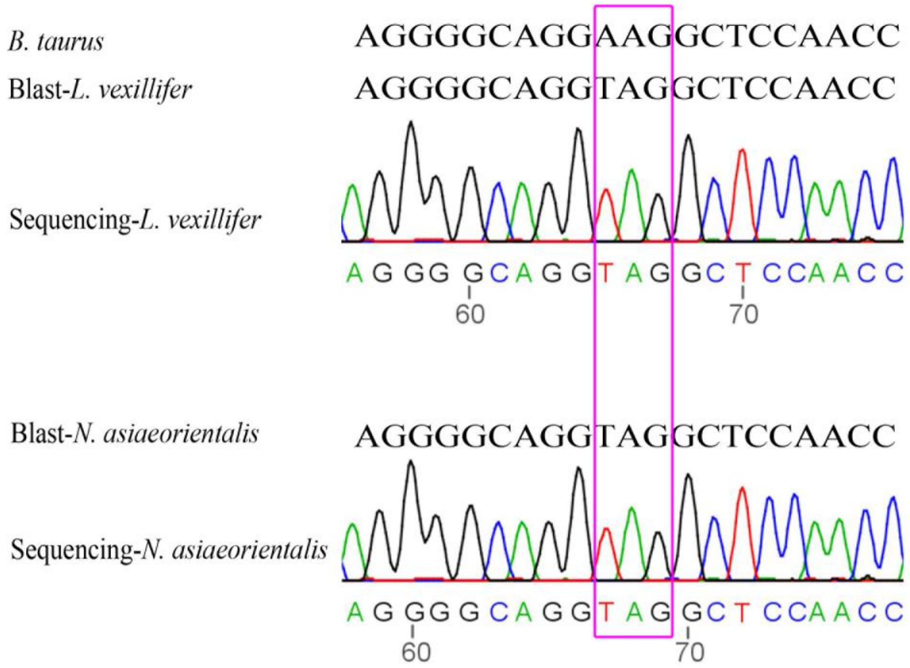

Identification of *ASIC4* gene of *L.vexillifer* and *N. asiaeorientalis.* The purplish red box indicates the stop codon.

References

1. Xue Y, Chidiac C, Herault Y, Gaveriaux-Ruff C: **Pain behavior in SCN9A (Nav1.7) and SCN10A (Nav1.8) mutant rodent models**. *Neuroscience Letters* 2021, **753**:135844.

2. Landy MA, Goyal M, Casey KM, Liu C, Lai HC: **Loss of Prdm12 during development, but not in mature nociceptors, causes defects in pain sensation**. *Cell Rep* 2021, **34**(13):108913-108913.
3. Liu Y, Liu Z, Wang K: **The Ca(2+)-activated chloride channel ANO1/TMEM16A: An emerging therapeutic target for epithelium-originated diseases?** *Acta Pharm Sin B* 2021, **11**(6):1412-1433.
4. McRoberts JW: **Cholecystokinin and pain: a review**. *Anesth Prog* 1986, **33**(2):87-90.
5. Yu L-N, Sun L-H, Wang M, Yan M: **Research progress of the role and mechanism of extracellular signal-regulated protein kinase 5 (ERK5) pathway in pathological pain**. *J Zhejiang Univ Sci B* 2016, **17**(10):733-741.
6. Obata K, Noguchi K: **MAPK activation in nociceptive neurons and pain hypersensitivity**. *Life Sciences* 2004, **74**(21):2643-2653.
7. Wang JW, Stifani S: **Roles of Runx Genes in Nervous System Development**. In: *RUNX Proteins in Development and Cancer.* Edited by Groner Y, Ito Y, Liu P, Neil JC, Speck NA, van Wijnen A. Singapore: Springer Singapore; 2017: 103-116.
8. Testa G, Cattaneo A, Capsoni S: **Understanding pain perception through genetic painlessness diseases: The role of NGF and proNGF**. *Pharmacological Research* 2021, **169**:105662.
9. Indo Y: **Genetics of congenital insensitivity to pain with anhidrosis (CIPA) or hereditary sensory and autonomic neuropathy type IV**. *Clinical Autonomic Research* 2002, **12**(1):I20-I32.
10. Coutelier M, Blesneac I, Monteil A, Monin M-L, Ando K, Mundwiller E, Brusco A, Le Ber I, Anheim M, Castrioto A *et al*: **A Recurrent Mutation in CACNA1G Alters Cav3.1 T-Type Calcium-Channel Conduction and Causes Autosomal-Dominant Cerebellar Ataxia**. *The American Journal of Human Genetics* 2015, **97**(5):726-737.
11. Davis JB, Gray J, Gunthorpe MJ, Hatcher JP, Davey PT, Overend P, Harries MH, Latcham J, Clapham C, Atkinson K *et al*: **Vanilloid receptor-1 is essential for inflammatory thermal hyperalgesia**. *Nature* 2000, **405**(6783):183-187.
12. Moore C, Gupta R, Jordt S-E, Chen Y, Liedtke WB: **Regulation of Pain and Itch by TRP Channels**. *Neurosci Bull* 2018, **34**(1):120-142.
13. Wirkner K, Sperlagh B, Illes P: **P2X3 Receptor Involvement in Pain States**. *Molecular Neurobiology* 2007, **36**(2):165-183.
14. Zhang W-j, Luo H-l, Zhu Z-m: **The role of P2X4 receptors in chronic pain: A potential pharmacological target**. *Biomedicine & Pharmacotherapy* 2020, **129**:110447.

15. Chauhan SA, Sahoo CG, Dikhit RM, Das P: **Acid-Sensing Ion Channels Structural Aspects, Pathophysiological Importance and Experimental Mutational Data Available Across Various Species to Target Human ASIC1**. *Current Drug Targets* 2019, **20**(1):111-121.

16. Wemmie JA, Taugher RJ, Kreple CJ: **Acid-sensing ion channels in pain and disease**. *Nature Reviews Neuroscience* 2013, **14**(7):461-471.

17. Donier E, Rugiero F, Jacob C, Wood JN: **Regulation of ASIC activity by ASIC4 - New insights into ASIC channel function revealed by a yeast two-hybrid assay**. *European Journal of Neuroscience* 2008, **28**(1):74-86.

18. Fu ES, Erasso DM, Zhuang GZ, Upadhyay U, Ozdemir M, Wiltshire T, Sarantopoulos KD, Smith SB, Maixner W, Martin ER *et al*: **Impact of human CA8 on thermal antinociception in relation to morphine equivalence in mice**. *Neuroreport* 2017, **28**(18):1215-1220.

19. Wang H, Heijnen CJ, van Velthoven CTJ, Willemen HLDM, Ishikawa Y, Zhang X, Sood AK, Vroon A, Eijkelkamp N, Kavelaars A: **Balancing GRK2 and EPAC1 levels prevents and relieves chronic pain**. *J Clin Invest* 2013, **123**(12):5023-5034.

20. Ozberk D, Haywood A, Sutherland HG, Yu C, Albury CL, Zunk M, George R, Good P, Griffiths LR, Hardy J *et al*: **Association of polymorphisms in ARRB2 and clinical response to methadone for pain in advanced cancer**. *Pharmacogenomics* 2022, **23**(5):281-289.

21. Imbrici P, Altamura C, Gualandi F, Mangiatordi GF, Neri M, De Maria G, Ferlini A, Padovani A, D'Adamo MC, Nicolotti O *et al*: **A novel KCNA1 mutation in a patient with paroxysmal ataxia, myokymia, painful contractures and metabolic dysfunctions**. *Molecular and Cellular Neuroscience* 2017, **83**:6-12.

22. Peng Y, Zhang Q, Cheng H, Yan G, Xing C: **Upregulation of ubiquitin conjugating enzyme E2B (Ube2b) ameliorates neuropathic pain by regulating Kcna2 (potassium voltage-gated channel subfamily A member 2) in primary afferent neurons**. *Bioengineered* 2021, **12**(1):7470-7480.

23. Noël J, Zimmermann K, Busserolles J, Deval E, Alloui A, Diochot S, Guy N, Borsotto M, Reeh P, Eschalier A *et al*: **The mechano-activated K+ channels TRAAK and TREK-1 control both warm and cold perception**. *The EMBO Journal* 2009, **28**(9):1308-1318.

24. Guo Y-P, Zhi Y-R, Liu T-T, Wang Y, Zhang Y: **Global Gene Knockout of Kcnip3 Enhances Pain Sensitivity and Exacerbates Negative Emotions in Rats**. *Frontiers in Molecular Neuroscience* 2019, **12**.

25. Mannerak MA, Lashkarivand A, Eide PK: **Trigeminal neuralgia and genetics: A systematic review**. *Molecular Pain* 2021, **17**:17448069211016139.

26. Ślęczkowska M, Almomani R, Marchi M, de Greef BTA, Sopacua M, Hoeijmakers JGJ,

Lindsey P, Salvi E, Bönhof GJ, Ziegler D *et al*: **Peripheral Ion Channel Gene Screening in Painful- and Painless-Diabetic Neuropathy**. *Int J Mol Sci* 2022, **23**(13).

27. Campa D, Gioia A, Tomei A, Poli P, Barale R: **Association of ABCB1/MDR1 and OPRM1 Gene Polymorphisms With Morphine Pain Relief**. *Clinical Pharmacology & Therapeutics* 2008, **83**(4):559-566.

28. Seo E-J, Efferth T, Panossian A: **Curcumin downregulates expression of opioid-related nociceptin receptor gene (OPRL1) in isolated neuroglia cells**. *Phytomedicine* 2018, **50**:285-299.

29. Ho KWD, Wallace MR, Staud R, Fillingim RB: **OPRM1, OPRK1, and COMT genetic polymorphisms associated with opioid effects on experimental pain: a randomized, double-blind, placebo-controlled study**. *The Pharmacogenomics Journal* 2020, **20**(3):471-481.

30. Nielsen LM, Christrup LL, Sato H, Drewes AM, Olesen AE: **Genetic Influences of OPRM1, OPRD1 and COMT on Morphine Analgesia in a Multi-Modal, Multi-Tissue Human Experimental Pain Model**. *Basic & Clinical Pharmacology & Toxicology* 2017, **121**(1):6-12.
